# Estimation of contemporary effective population size in plant populations: Limitations of genomic datasets

**DOI:** 10.1111/eva.13691

**Published:** 2024-05-03

**Authors:** Roberta Gargiulo, Véronique Decroocq, Santiago C. González‐Martínez, Ivan Paz‐Vinas, Jean‐Marc Aury, Isabelle Lesur Kupin, Christophe Plomion, Sylvain Schmitt, Ivan Scotti, Myriam Heuertz

**Affiliations:** ^1^ Royal Botanic Gardens, Kew Richmond UK; ^2^ INRAE Univ. Bordeaux, UMR 1332 BFP Villenave d'Ornon France; ^3^ INRAE Univ. Bordeaux Cestas France; ^4^ Department of Biology Colorado State University Fort Collins Colorado USA; ^5^ CNRS, ENTPE, UMR5023 LEHNA Université Claude Bernard Lyon 1 Villeurbanne France; ^6^ Génomique Métabolique, Genoscope, Institut François Jacob, CEA, CNRS, Univ Evry Université Paris‐Saclay Evry France; ^7^ AMAP Univ. Montpellier, CIRAD, CNRS, INRAE, IRD Montpellier France; ^8^ INRAE, URFM Avignon France

**Keywords:** conservation genomics, effective population size, GONE, linkage disequilibrium, plants

## Abstract

Effective population size (*N*_e_) is a pivotal evolutionary parameter with crucial implications in conservation practice and policy. Genetic methods to estimate *N*_e_ have been preferred over demographic methods because they rely on genetic data rather than time‐consuming ecological monitoring. Methods based on linkage disequilibrium (LD), in particular, have become popular in conservation as they require a single sampling and provide estimates that refer to recent generations. A software program based on the LD method, GONE, looks particularly promising to estimate contemporary and recent‐historical *N*_e_ (up to 200 generations in the past). Genomic datasets from non‐model species, especially plants, may present some constraints to the use of GONE, as linkage maps and reference genomes are seldom available, and SNP genotyping is usually based on reduced‐representation methods. In this study, we use empirical datasets from four plant species to explore the limitations of plant genomic datasets when estimating *N*_e_ using the algorithm implemented in GONE, in addition to exploring some typical biological limitations that may affect *N*_e_ estimation using the LD method, such as the occurrence of population structure. We show how accuracy and precision of *N*_e_ estimates potentially change with the following factors: occurrence of missing data, limited number of SNPs/individuals sampled, and lack of information about the location of SNPs on chromosomes, with the latter producing a significant bias, previously unexplored with empirical data. We finally compare the *N*_e_ estimates obtained with GONE for the last generations with the contemporary *N*_e_ estimates obtained with the programs *currentNe* and NeEstimator.

## 1 INTRODUCTION

Effective population size (*N*_e_) is an evolutionary parameter introduced by Wright (1931), which determines the rate of genetic change due to genetic drift and is therefore linked with inbreeding and loss of genetic variation in populations, including adaptive potential (Franklin, 1980; Jamieson & Allendorf, 2012; Waples, 2022). The importance of contemporary effective population size in conservation biology is increasingly recognized, and the concept implemented in conservation practice (Frankham et al., 2014; Luikart et al., 2010; Montes et al., 2016) and policy (Graudal et al., 2014; Hoban et al., 2013; Kershaw et al., 2022; O'Brien et al., 2022). For example, *N*_e_ has been included as a headline genetic indicator to support Goal A and Target 4 of the Kunming‐Montreal Global Biodiversity Framework of the UN's Convention on Biological Diversity (CBD, 2022), as the proportion of populations within species with *N*_e_ > 500, that are expected to have sufficient genetic diversity to adapt to environmental change (Hoban et al., 2020; Jamieson & Allendorf, 2012).

Contemporary *N*_e_ can be estimated using demographic or genetic methods (Felsenstein, 2019; Luikart et al., 2010; Wang et al., 2016; Waples, 2016; Wright, 1969). Demographic estimators require detailed ecological observations over time for the populations of interest (Felsenstein, 2019; Nunney, 1993; Wright, 1969), which is not necessary for genetic estimators (Wang et al., 2016; Waples, 2016). Methods that can provide *N*_e_ estimates based on a single sampling point in time (Wang, 2016) have become particularly popular, especially in studies focused on species for which budget and time allocated are limited, elusive species that are difficult to track and monitor (Luikart et al., 2010), and species for which information about distribution is scarce. The current biodiversity crisis and the limited resources for conservation have recently fuelled the development and application of *N*_e_ estimators that rely on cost‐effective, non‐genetic proxy data across a wide range of species of conservation concern (Hoban et al., 2020; Hoban, Bruford, et al., 2021). Population census size, *N*_C_, has been used to infer *N*_e_ when genetic *N*_e_ estimates are not available, relying on the ratio *N*_e_/*N*_C_ = 0.1 (where *N*_C_ is the adult census size of a population) (Frankham et al., 2014; Hoban, Paz‐Vinas, et al., 2021; Palstra & Fraser, 2012). This rule‐of‐thumb ratio is pragmatic for conservation (but see Fady & Bozzano, 2021), as shown in application tests in different countries for different species of conservation concern (Hoban et al., 2023; Thurfjell et al., 2022). However, research needs to progress to better understand *N*_e_ estimation methods and potential deviations from the ratio *N*_e_/*N*_C_ = 0.1, which are expected for example across populations within species or in species with life‐history traits that favour individual persistence (Frankham, 2021; Gargiulo et al., 2023; Hoban et al. 2020; Hoban, Paz‐Vinas, et al., 2021; Jamieson & Allendorf, 2012; Laikre et al., 2021). Current genetic estimators of contemporary *N*_e_ work well in small and isolated populations, which match many populations of conservation concern, but they are difficult to apply in species with a large and continuous distribution (Fady & Bozzano, 2021; Santos‐del‐Blanco et al., 2022). In such species, genetic isolation by distance, overlapping generations, and difficulty to define representative sampling strategies can affect the accuracy of estimates of *N*_C_, *N*_e_ and their ratio (Neel et al., 2013; Nunney, 2016; Santos‐del‐Blanco et al., 2022). Plant species embody some of the features mentioned above, as they often have complex life‐history traits (e.g., overlapping generations, long lifespans), reproductive systems (i.e., mixed clonal and sexual reproduction, mixed selfing and outcrossing strategies) and continuous distribution ranges (De Kort et al., 2021; Petit & Hampe, 2006). Therefore, they are particularly interesting to help improve our understanding of *N*_e_ estimation methods.

Genetic drift generates associations between alleles at different loci, known as linkage disequilibrium (LD), at a rate inversely proportional to *N*_e_ (Hill, 1981; Waples et al., 2016). LD between loci can be used to obtain a robust estimate of contemporary *N*_e_ from genetic data at a single time point, and this explains the popularity of the LD method compared to the earlier developed two‐sample temporal methods (Luikart et al., 2010; Waples, 2024) and the development of numerous tools for the estimation of LD*N*_e_ from genetic and genomic data (Barbato et al., 2015; Do et al., 2014; Santiago et al., 2020; Wang et al., 2016). The *N*_e_ estimates obtained with the LD method generally refer to a few generations back in time (Do et al., 2014; Luikart et al., 2010) and, depending on the genetic distances between loci, it is possible to obtain *N*_e_ at different times in the past (Santiago et al., 2024; see also the review on timescales of *N*_e_ estimates in Nadachowska‐Brzyska et al., 2022). In particular, LD between closely linked loci can be used to estimate *N*_e_ over the historical past (Barbato et al., 2015; Do et al., 2014; Hayes et al., 2003; Qanbari et al., 2010; Santiago et al., 2020; Sved, 1971; Wang et al., 2016), whereas loosely linked or unlinked loci can be used to estimate *N*_e_ in the recent past (Novo, Ordás, et al., 2023; Novo, Pérez‐Pereira, et al., 2023; Qanbari, 2019; Sved et al., 2013; Wang et al., 2016; Waples, 2006a; Waples & Do, 2008). However, as other methods to estimate *N*_e_, the LD method is not devoid of biases and drawbacks, mostly relating to the assumption that the population is isolated, which is rarely satisfied (England et al., 2010; Hill, 1981; Waples, 2024; Waples & England, 2011), and to the occurrence of age structure (Hössjer et al., 2016; Nunney, 1991; Robinson & Moyer, 2013; Ryman et al., 2019; Waples et al., 2014; Waples & Do, 2010; Yonezawa, 1997).

In this study, we aimed to explore the limitations of plant genomic datasets when estimating contemporary *N*_e_. We mostly focused on estimating *N*_e_ using the software program GONE (Santiago et al., 2020), but we also provide *N*_e_ estimates obtained with NeEstimator (Do et al., 2014) and the recently developed program, *currentNe* (Santiago et al., 2024). These programs provide recent historical and contemporary *N*_e_ estimates, respectively, using the LD method, though they differ mostly in the data requirement and timescales of estimates provided. GONE is capable of exploiting the full range of LD among loci in a dataset, therefore providing *N*_e_ estimates that are reliable up to 200 generations ago; NeEstimator and *currentNe* provide *N*_e_ estimates that represent the average over a few recent generations, and the number of generations representing an estimate increases with the number of chromosomes of the species (Santiago et al., 2024).

We explored the technical requirements of GONE by conducting power analyses aimed at testing how the number of SNPs, the proportion of missing data, the number of individuals, the lack of information about the location of SNPs on chromosomes, and the occurrence of population structure might affect *N*_e_ estimation. The *N*_e_ estimates obtained with GONE were then compared to the ones obtained with NeEstimator and *currentNe*, and discussed in light of the biological and ecological features of the species. Our findings help better understand the limitations and potentialities of genomic datasets when estimating LD‐based, one‐sample *N*_e_, providing new insights on how to use current methods.

## 2 METHODS

### 2.1 Datasets

We selected four datasets obtained with different high‐throughput sequencing techniques from different plant taxa (*Symphonia globulifera* L. f. (Clusiaceae), *Mercurialis annua* L. (Euphorbiaceae), *Fagus sylvatica* L. (Fagaceae), *Prunus armeniaca* L. (Rosaceae)), to represent different botanical groups, ecosystems, generation times and reproductive strategies. Sampling strategies in the datasets encompassed different sample sizes for markers and individuals, and datasets featured distinct levels of population genetic structure (Table 1).

**TABLE 1:** Details of the different plant genomic datasets analyzed in the present study.

Species name	Life‐form	Reproductive system of populations analyzed	Gene pools (#samples)	Data type	Average frequency of missing data per individual	#chromosomes/scaffolds/contigs analyzed in GONE	Average #SNPs per scaffold or chromosome^a^	Total #SNPs^b^	Reference	Issues explored (affecting *N*_e_ estimation in GONE)
*Symphonia globulifera* L. f.	Perennial (tree)	Monoecious, mixed mating with predominant outcrossing (Degen et al., 2004)	Species 1 (228) Species 2 (107) Species 3 (30)	Targeted sequence capture	0.04	125 (contigs)	247	30,863	Schmitt et al. (2021)	Minimum number of SNPs required
*Mercurialis annua* L.	Annual	Various mating systems, analyses based on dioecious populations; obligate outcrosser (González‐Martínez et al., 2017)	Atlantic (12) Core (16) Mediterranean (12)	Targeted gene (exome) capture	0.01	48 (contigs)	670	32,151	González‐Martínez et al. (2017)	Influence of sample size
*Fagus sylvatica* L.	Perennial (tree)	Monoecious, predominant outcrossing (Merzeau et al., 1994)	Mt. Ventoux, France (167)	Whole genome sequencing	0.81 (with 27 scaffolds)	12–150 (scaffolds)	~470 K (with 27 scaffolds)	~13 M (with 27 scaffolds)	See data availability section	Influence of missing data
*Prunus armeniaca* L.	Perennial (tree)	Monoecious, self‐incompatible (Groppi et al., 2021)	Southern (56) Northern (199) (see Table S1)	Whole genome sequencing	0.07	8 (chromosomes)	~3 M (440 K)	~24 M (3.5 M in the subsampled dataset)	Groppi et al. (2021)	Influence of number of SNPs, of missing data, of sample size, of population structure, of using scaffolds instead of chromosomes

^a^ In the map file, number of lines divided by number of scaffolds/chromosomes;

^b^ Number of lines in the map file.

For boarwood, *S. globulifera* s.l., a widespread and predominantly outcrossing evergreen tree typical of mature rainforests in Africa and the Neotropics (Degen et al., 2004; Torroba‐Balmori et al., 2017), we used the targeted sequence capture dataset described in Schmitt et al. (2021). Three sympatric gene pools were identified in a lowland forest in French Guiana, likely corresponding to three biological species, described as *Symphonia* sp. 1, *Symphonia* sp. 2 and *Symphonia* sp. 3 (Schmitt et al., 2021). To avoid the influence of admixture on the estimation of *N*_e_, we first divided the dataset in three subsets based on the analysis of genetic structure performed in the software Admixture v1.3.0 (see Schmitt et al., 2021), selecting only the individuals with a *Q*‐value (cluster membership coefficient) ≥ 95% to each of the three genetic clusters (Species 1, Species 2 and Species 3; File S1). We then selected the 125 genomic scaffolds with the largest number of SNPs (see Table 1).

For the annual mercury, *M. annua*, an annual plant with variable mating systems (monoecious, dioecious, androdioecious), ploidy levels (2×, 4×–12×) (Obbard, Harris, Buggs, & Pannell, 2006; Obbard, Harris, & Pannell, 2006), potential to produce seed banks, and typical of open or disturbed habitats in Europe and North Africa, we used the gene capture dataset described in González‐Martínez et al. (2017), obtained from 40 diploid dioecious individuals grown from seeds, representative of 10 localities and three main gene pools in the species (as described after the fastStructure analysis in González‐Martínez et al., 2017). We selected the 48 scaffolds with the largest number of SNPs and ran the analyses by considering each gene pool separately: (1) ancestral populations from Turkey and Greece (“Core”), (2) range‐front populations from northeastern Spain (“Mediterranean”), or (3) range‐front populations from northern France and the UK (“Atlantic”) (see Table 1).

For the common beech, *F. sylvatica*, a deciduous predominantly outcrossing tree of European temperate forests (Merzeau et al., 1994), we analyzed genomic scaffolds from a single, contiguous stand (plot N1; Oddou‐Muratorio et al., 2021) within a relatively isolated French population (Mt. Ventoux, southeastern France, *N*_C_ ≃ hundreds of thousands, also depending on the gene flow range), in which population genetic structure is neither observed nor expected (Csilléry et al., 2014). Mapping of short‐reads paired Illumina sequences was independently performed for each one of the 167 individuals of the population against the genome assembly (available at www.genoscope.cns.fr/plants) using bwa‐mem2 2.0 (Li & Durbin, 2009). SNPs were first called using GATK 3.8 (Van der Auwera & O'Connor, 2020) using the following parameters: ‐nct 20 ‐variant_index_type LINEAR variant_index_parameter 128,000. SNPs were also called using samtools v1.10/bcftools v1.9 (Danecek et al., 2021) with default parameters. Following these two SNPs calling steps, we performed a three‐steps filtering process: (i) only diallelic SNPs were kept, (ii) the minimum allele frequency (MAF, upper case used at the individual level), calculated on the basis of all the reads containing the SNP, was set to 30% (note that GONE does not require the application of MAF filtering, and such filtering might cause a small upward bias in the estimation), (iii) individual genotypes with sequencing depth less than 10 were recoded into «./.» meaning that both alleles are missing. We then identified SNPs found by both GATK and samtools using the ‐ diff flag of vcftools v0.1.15 with tabix‐0.2.5 (Danecek et al., 2011). A nucleotide polymorphism was considered to be a SNP if at least one individual was found to be heterozygous at the position. On average, for each individual, 88.5% of the sequencing reads mapped properly onto the assembly. The final VCF contained 18,192,174 variants, and is available at the Portail Data INRAe (https://doi.org/10.57745/FJRYI1).

We re‐ordered the 406 genomic scaffolds available based on their number of SNPs, and selected 150 scaffolds with the largest number of SNPs. We tested different combinations of input subsets, with numbers of scaffolds ranging from 12 to 150 (provided that SNPs per scaffold <1 million and total number of SNPs <10 million, see the requirements of GONE below), and numbers of individuals ranging from 5 to 167 (the total sample size).

For the apricot, *P. armeniaca*, we estimated *N*_e_ using whole genome resequencing data (21× depth of coverage by ILLUMINA technology) for wild Central Asian, self‐incompatible populations of the species (Groppi et al., 2021). Variant sites were mapped to the eight chromosomes of the species and ranged between 2.3 and 6.2 million per chromosome (total number of variant sites: 24 M). As these exceeded the total number allowed in GONE, we downsampled the number of SNPs prior to the analyses. We also analyzed the datasets by considering the different gene pools recovered in Groppi et al. (2021) (see Supp. Fig. S20 in Groppi et al. 2021), namely the Southern (red cluster) and Northern (yellow cluster) gene pools, as obtained with fastStructure (Raj et al., 2014) (see next subsection).

### 2.2 Data analyses in GONE

#### 2.2.1 Analyses for all species

We performed *N*_e_ estimation with the software GONE (Santiago et al., 2020). GONE generates contemporary or recent historical estimates of *N*_e_ (i.e., in the 100–200 most recent generations) using the LD method. GONE uses linkage information represented by mapped SNPs, ideally mapped to chromosomes. Chromosome mapping is rarely available for non‐model species, and in our case was only fully available for the apricot (*P. armeniaca*) dataset. In the absence of chromosome mapping information for the other species, we treated genomic scaffolds as chromosomes. In terms of requirements, GONE accepts a maximum number of chromosomes of 200 and a maximum number of SNPs of 10 million, with a maximum number of SNPs per chromosome of 1 million, although the program uses up to 50,000 random SNPs per chromosome for the computations when the total number of SNPs is larger. A complete workflow of the analyses carried out in GONE is available at https://github.com/Ralpina/Ne‐plant‐genomic‐datasets (Gargiulo, 2023); the input parameter file used for the final analyses is available in File S2.

#### 2.2.2 Influence of missing data on *N*_e_ estimation

The influence of missing data on *N*_e_ estimation in GONE was evaluated using the dataset from *F. sylvatica*. After keeping 67 individuals with less than 95% missing data, we permuted individuals (without replacement) to generate 150 datasets of 35 individuals, and estimated *N*_e_ in GONE for each dataset. Proportion of missing data per individual for each permuted dataset was calculated in vcftools v0.1.16 (Danecek et al., 2011) from an average of ~25%–95%; results were plotted in R v4.2.2 (R Core Team, 2019). In addition, we used the dataset of *P. armeniaca* to evaluate how *N*_e_ changed when manually introducing missing data. We selected all individuals from the Northern gene pool with a *Q*‐value (cluster membership coefficient) ≥ 99% (77 individuals) to rule out the influence of admixture, and replaced some of the individual genotypes with missing values using a custom script (available at: https://github.com/Ralpina/Ne‐plant‐genomic‐datasets). We generated two datasets with a proportion of missing data per individual of 20% and 40%, respectively, and then computed *N*_e_ in GONE for each dataset obtained.

#### 2.2.3 Influence of number of SNPs on *N*_e_ estimation

The influence of the number of SNPs on *N*_e_ estimation in GONE was evaluated using the dataset of *P. armeniaca*. From the Northern gene pool, we first selected the individuals with a *Q*‐value ≥99% to rule out the influence of admixture. We drew random subsets of variant sites (without replacement) including 40 K, 80 K, 150 K, 300 K, 500 K, 3.5 M, 7 M, and 10 M SNPs, respectively, and generated 50 replicates for each subset; we then estimated *N*_e_ in GONE for each subset and obtained the geometric mean and the 95% confidence intervals across the 50 replicate subsets with the same number of SNPs (using the functions *exp(mean(log(x)))* and *quantile* in R).

#### 2.2.4 Influence of the sample size on *N*_e_ estimation

We used the Northern gene pool of *P. armeniaca* to assess how *N*_e_ estimates changed depending on the number of samples considered and the uncertainty associated with individual sampling. We first downsampled the number of SNPs to 3.5 M (to satisfy GONE requirements), and varied the sample sizes included in the analyses from 15 to 75 (i.e., approx. the total number of individuals of the Northern gene pool with a *Q*‐value ≥ 99%). For each sample size group, we generated 50 subsets (without replacement within the subset) of individuals and estimated *N*_e_ in GONE for each subset; we then estimated the geometric mean and the 95% confidence intervals across subsets with the same sample size (using the functions *stat_summary(fun. data = median_hilow, fun.args = list(conf.int = 0.95)* and *stat_summary(fun = “geometric.mean”* (psych package) in R).

#### 2.2.5 Influence of population admixture on *N*_e_ estimation

We also evaluated how genetic structure within gene pools influenced *N*_e_ estimation in GONE for both the Southern and Northern gene pools of *P. armeniaca*. We first downsampled the number of SNPs to 3.5 M to satisfy GONE requirements, as described above. We then distributed the individuals of each gene pool into five (overlapping) subsets based on individual *Q*‐values (lower bounds of 70%, 80%, 90%, 95%, and 99%), resampled individuals (without replacement) in each *Q*‐value subset 50 times, standardizing sample sizes to the sample size of the smallest *Q*‐value subset within a gene pool (i.e., 21 individuals as in the 99% *Q*‐value subset of the Southern gene pool and 77 individuals as in the 99% *Q*‐value subset of the Northern gene pool, see Table S1 for the original sample sizes). We then estimated *N*_e_ in GONE and obtained 95% confidence intervals across the 50 resampled datasets of the same *Q*‐value subset within a gene pool (using the R function *stat_summary* mentioned above). We also combined all individuals from the two gene pools (255 individuals), resampled either 22 or 77 individuals 50 times without replacement, and estimated *N*_e_ in GONE and the related confidence intervals as explained above, to evaluate the effect of missing the two gene pools on the *N*_e_ estimates obtained.

#### 2.2.6 Effect of using genomic scaffolds rather than chromosomes

We evaluated the effect of using genomic scaffolds to estimate linkage groups when chromosome information is not available. Using the downsampled dataset of 3.5 M SNPs from *P. armeniaca*, we selected from the Northern gene pool 45 random individuals with a *Q*‐value ≥ 99%, to rule out the influence of admixture. For this dataset, five different chromosome maps were then created, progressively assigning SNPs to 8 (true value), 16, 32, 64 and 128 chromosomes (as if they were genomic scaffolds, see script and related explanation at https://github.com/Ralpina/Ne‐plant‐genomic‐datasets#4‐effect‐of‐using‐genomic‐scaffolds‐instead‐of‐chromosomes‐on‐ne‐estimation). We then estimated *N*_e_ in GONE using five corresponding chromosome map files and keeping the same ped (genotypes) file.

### 2.3 Data analyses in NeEstimator

We also used the LD method as implemented in the software NeEstimator v2 (Do et al., 2014) to estimate the *N*_e_ of our populations. NeEstimator uses unmapped SNP information and assumes that SNPs are independently segregating (typically, SNPs at short physical distances, for example those in the same short genomic scaffolds or loci, are filtered previous to the analysis, see below). Therefore, it provides an *N*_e_ estimate based on the LD generated by random genetic drift, which reflects *N*_e_ in very recent generations (Waples et al., 2016). However, accuracy and precision will be both affected by (1) the assumption of independent segregation in genomic datasets, as SNPs are necessarily packed on a limited number of chromosomes and thus they provide non‐independent information, and especially (2) the occurrence of overlapping pairs of loci, each locus appearing in multiple pairwise comparisons (i.e., two aspects of the issue known as pseudoreplication; Purcell et al., 2007; Waples, 2024; Waples et al., 2016, 2022). Although the influence of this issue on bias and precision is difficult to address completely, some bias corrections have been proposed, for example applying a correction based on the genome size of the species being analyzed (formula in Waples et al., 2016), restrict comparisons to pairs of loci occurring on different chromosomes (Waples, 2024), or using only one SNP per scaffold or thinning scaffolds based on discrete window sizes (Purcell et al., 2007). To correct the bias due to physical linkage, we therefore applied the correction in Waples et al. (2016), by dividing the *N*_e_ estimates obtained by *y =* 0.098 + 0.219 *×* ln(*Chr*), where *Chr* is the haploid number of chromosomes, when information about the number of chromosomes was available.

As low‐frequency alleles upwardly bias *N*_e_, we followed the recommendations in Waples (2024) and excluded singleton alleles (Waples, 2024; Waples & Do, 2010). We also ran the analyses without applying a filter for rare alleles, to be able to compare the results obtained with NeEstimator with those from GONE and *currentNe*. Confidence intervals were obtained via jackknifing over samples (Do et al., 2014; Jones et al., 2016). As NeEstimator cannot handle very large datasets (with >100,000 loci, see https://www.molecularfisherieslaboratory.com.au/neestimator‐software/), we reduced the number of SNPs in the *F. sylvatica* and *P. armeniaca* datasets by randomly subsampling 50,000 SNPs across chromosomes.

### 2.4 Data analyses in *currentNe*

We used the newly developed software program *currentNe* (Santiago et al., 2024) to obtain contemporary *N*_e_ estimates that are directly comparable to the ones obtained with NeEstimator (referring to the most recent generations in the past). The practical advantages of *currentNe* are the possibility to include thousands of SNPs in the analyses (with an upper limit of 2 million loci), the lack of a minor allele frequencies requirement, and the lower computational effort. Moreover, the program produces confidence intervals around *N*_e_ based on artificial neural networks, can accommodate complex mating systems and is accurate with small sample sizes (Santiago et al., 2024). *CurrentNe* produces two types of estimation, depending on whether SNPs mapping is available (*N*_e_ estimation based on LD between chromosomes) or not (*N*_e_ estimation by integration over the whole genome). In the latter case, the program assumes that each of the given chromosomes is about 1 Morgan long. When the number of chromosomes is unknown, the mapping of SNPs to scaffolds might also be used for the first estimation type (based on LD between “chromosomes”). However, scaffolds might be much shorter than chromosomes, and SNPs will not be totally independent (as scaffolds might actually belong to the same chromosome). Therefore, we estimated *N*_e_ in *currentNe* for all the species included in our study except *S. globulifera* s.l., as the number of chromosomes was not available for the species.

## 3 RESULTS AND DISCUSSION

### 3.1 Data analyses in GONE

Our study explores the limitations associated with genomic datasets when estimating *N*_e_ using the LD method as implemented in the program GONE, and compares estimates of recent historical *N*_e_ obtained with GONE with estimates of contemporary *N*_e_ as obtained with NeEstimator and *currentNe*. Below, we will first focus on the limitations of plant genomic datasets as explored using the software GONE and then discuss the differences observed when *N*_e_ was calculated using GONE, NeEstimator, and *currentNe*.

One limitation usually associated with reduced representation sequencing datasets is the short length of the reads or scaffolds. We tested how this limitation would influence *N*_e_ estimation in GONE using the datasets of *S. globulifera* and *M. annua*. *N*_e_ estimation in GONE failed for the three biological species of *S. globulifera*, as the program returned the error “too few SNPs” for each of the three species datasets. This was caused by the relatively small number of SNPs per scaffold (averaging ≃250 SNPs) and, in turn, by the relatively short length of the scaffolds (length ranging from 5421 to 931 positions) which prevented GONE from producing reliable *N*_e_ estimates. *N*_e_ estimates were instead obtained for *M. annua*, whose average number of SNPs per contig was 670 (Table 1).

#### 3.1.1 Influence of missing data on *N*_e_ estimation

**FIGURE 1:**
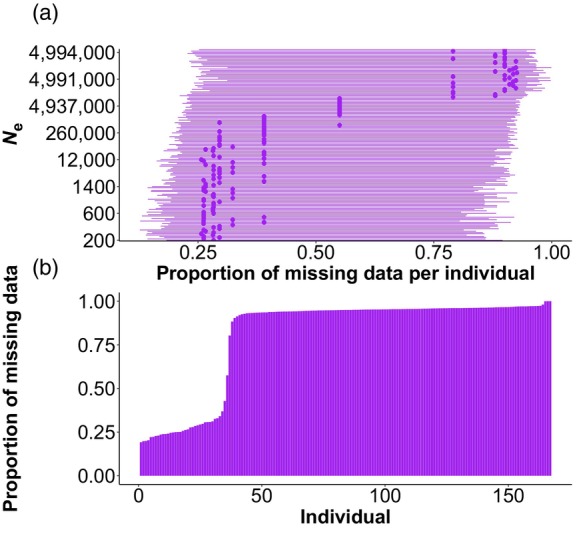
In (a), ranked *N*_e_ estimates in the most recent generation in 150 datasets of 35 individuals with different proportions of missing data (excluding individuals with a proportion of missing data >0.95) of *Fagus* s*ylvatica*; ranges represent standard deviations for the proportion of missing data per individual, whereas points represent median values over 150 datasets. Analyses based on the dataset with the 27 genomic scaffolds with the largest number of SNPs (excluding the scaffolds with >1 M SNPs). In (b), proportion of missing data per individual in the complete dataset of *F. sylvatica*.

**FIGURE 2:**
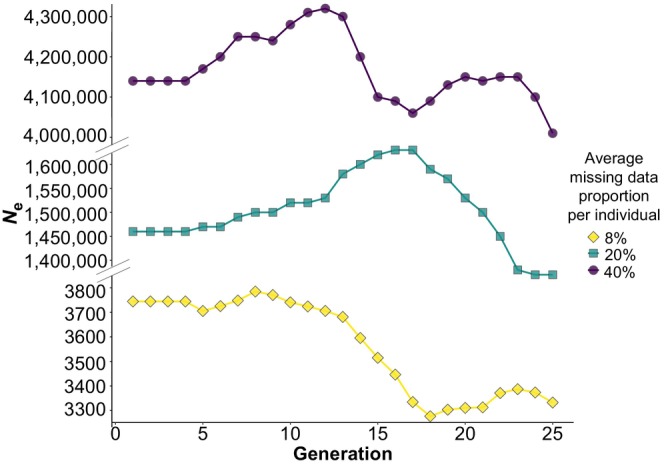
Influence of missing data on *N*_e_ estimation in GONE. Missing genotypes were manually introduced into the dataset of *Prunus armeniaca*, generating pseudo‐genotypes with an average proportion of missing data ranging from 20% to 40%. The original dataset is shown for comparison (missing data = 8%). Note the different *y*‐scales in the three facets.

The effect of missing data on *N*_e_ estimation is evident from the results obtained when analysing the dataset of *F. sylvatica*, and from the results obtained when analysing the dataset of *P. armeniaca* in which genotype data were manually excluded. For *F. sylvatica*, 35 individuals had a proportion of missing data <50% (Figure 1b). Increasing the proportion of missing data in the permuted datasets of 35 individuals produced an acute increase in the *N*_e_ estimates obtained with GONE (see Figure 1a); for instance, increasing the median proportion of missing data per individual from 25% to 35% produced *N*_e_ estimates increasing from 200 to a few millions. Likewise, when missing data proportion per individual of *P. armeniaca* increased above 20%, we obtained *N*_e_ estimates that were > 350 times larger than those obtained from the original dataset (average missing data proportion per individual ≃8%) (Figure 2). This relationship between missing data and *N*_e_ estimates is consistent with what was previously found (e.g., Marandel et al., 2020), although the loss of accuracy in the *N*_e_ estimation is extreme and suggests that either individuals with >20% missing data should be removed from the dataset before estimating *N*_e_ or SNPs with missing data in a given percentage of individuals (e.g., 50% by default assumed by GONE) should be removed, provided that the dataset includes a sufficient number of SNPs. However, in species with large *N*_e_, reducing the sample size (S) to a number ≪ true *N*_e_ introduces further uncertainties in the *N*_e_ estimation using the LD method, regardless of the number of loci used (Marandel et al., 2019; Waples, 2024), in addition to the sampling error already expected because of the finite sample size (e.g., Peel et al., 2013).

#### 3.1.2 Influence of number of SNPs on *N*_e_ estimation

**FIGURE 3:**
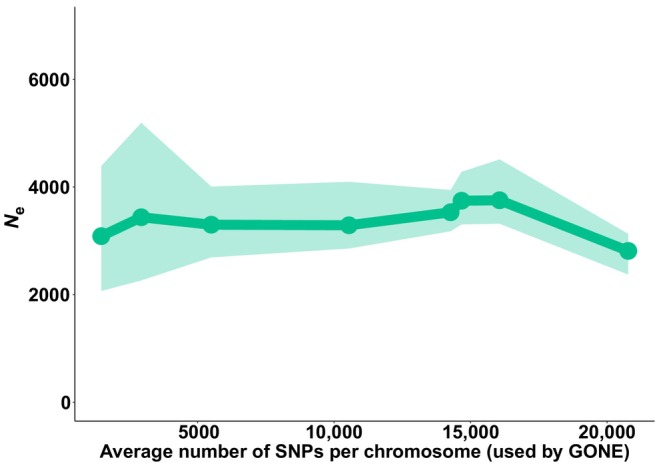
*N*_e_ estimates obtained with GONE over the most recent generation for the Northern gene pool of *Prunus armeniaca* as a function of the number of SNPs. Points represent the geometric mean values across 50 replicates; shaded area represents 95% confidence intervals across replicates. Note that GONE uses a maximum of 50,000 SNPs per chromosome, even if provided with a larger number (with 1 million per chromosome being the maximum number accepted); the number of SNPs in each of the eight subsets analyzed ranged from 10^4^ to 10^7^, corresponding to a range of ≃5000 to ≃20,000 SNPs per chromosome used by GONE.

The influence of the number of SNPs per chromosome was explored using the dataset from *P. armeniaca* (Northern gene pool), which was the only dataset with SNPs fully mapped to chromosomes. Increasing the number of SNPs per chromosome affected point *N*_e_ estimates only slightly, and influenced the apparent precision of the estimates more obviously, especially for a total number of SNPs above 300,000, corresponding to an average of 10,000 SNPs per chromosome of *P. armeniaca* used by GONE (Figure 3). Accuracy and precision of *N*_e_ estimates based on LD are expected to be affected by two types of pseudoreplication: (1) the non‐independent information content provided by thousands of linked SNPs, and especially (2) the occurrence of overlapping pairs of loci, each locus appearing multiple times in pairwise comparisons (Waples et al., 2016, 2022). Therefore, the narrower confidence intervals we obtained when increasing the number of SNPs are partially due to the inclusion of overlapping pairs of loci for the *N*_e_ estimation, which artificially increases the degrees of freedom that make CIs tight. The drop in the *N*_e_ geometric mean value associated with the dataset with >20,000 SNPs might be due to the inclusion of more physically linked SNPs, but it might also be due to the uncertainty associated with the specific SNPs included in the analysis.

For practical purposes, our results in *P. armeniaca* show that adding more than 2000 SNPs per chromosome, with a large sample size (>75), does not substantially improve the accuracy and the precision of the estimation, in line with what is shown in previous studies focusing on LD*N*_e_ (Marandel et al., 2020). We have not explored whether using fewer SNPs in this dataset would significantly affect accuracy and precision, and it is possible that *N*_e_ estimates would remain consistent even if using <2000 SNPs per chromosome.

Santiago et al. (2020) noted that the accuracy of the estimation is proportional to the sample size and to the square root of SNPs pairs, and therefore researchers might partially compensate for small sample sizes by increasing the number of SNPs. However, as the information content of a dataset depends on the amount of recombination and on the pedigree of the individuals included in the analyses, an estimation based on a small number of samples will not necessarily be representative of the entire population, especially if *N*_e_ is large (King et al., 2018; Santiago et al., 2020; Waples, 2024). Furthermore, the marginal benefit of increasing the number of SNPs beyond tens of thousands is counterbalanced by poor precision if CIs are generated using incorrect degrees of freedom, which is often the case with thousands of non‐independent SNPs (Do et al., 2014; Jones et al., 2016; Luikart et al., 2021; Moran et al., 2019; Waples et al., 2022). Finally, Waples (2024) also points out that adding more than a few thousand SNPs increases the precision only slightly and is more beneficial when the true *N*_e_ is large.

#### 3.1.3 Influence of the sample size on *N*_e_ estimation

**FIGURE 4:**
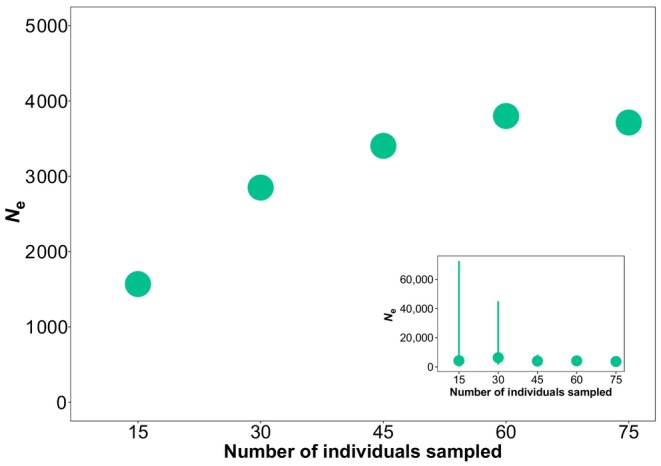
Change in the *N*_e_ estimates as a function of the sample size in *Prunus armeniaca* (Northern gene pool). Points represent geometric means across subsets of individuals, sampled without replacement 50 times. The insert also shows 95% confidence intervals (point ranges) estimated over the 50 replicate subsets.

We evaluated the influence of the sample size using the Northern gene pool of *P. armeniaca*. Increasing sample sizes to over thirty samples led to more consistent *N*_e_ estimates and reduced the chances of obtaining *N*_e_ estimates only representative of a few individual pedigrees (Figure 4), as previously observed when using the LD method (Antao et al., 2011; Marandel et al., 2019; Nunziata & Weisrock, 2018; Palstra & Ruzzante, 2008; Santiago et al., 2020; Tallmon et al., 2010; Waples et al., 2016; Waples & Do, 2010). Including in the *N*_e_ estimation a number of samples that is representative of the true *N*_e_ of the population is crucial in large populations, where the genetic drift signal in recent generations is weak (Barbato et al., 2015; Do et al., 2014; Luikart et al., 2010; Palstra & Ruzzante, 2008; Santiago et al., 2020; Wang et al., 2016; Waples, 2024). On the contrary, small populations experience more genetic drift, and therefore the LD method is particularly powerful in such populations. Estimates of *N*_e_ remain small in small populations even with larger sample sizes, hence the important conservation implication that small populations cannot be mistaken for large populations (Santiago et al., 2020; Waples et al., 2016; Waples & Do, 2010). For the Northern gene pool of wild apricots, we obtained an *N*_e_ estimate <2000 when the sample size was equal to 15, and progressively obtained higher values increasing up to a plateau of *N*_e_ ≃ 4000, for larger sample sizes. This confirms the expectation that a large sample size is needed to estimate a large *N*_e_ (Antao et al., 2011; Tallmon et al., 2010).

#### 3.1.4 Influence of admixture on *N*_e_ estimation

**FIGURE 5:**
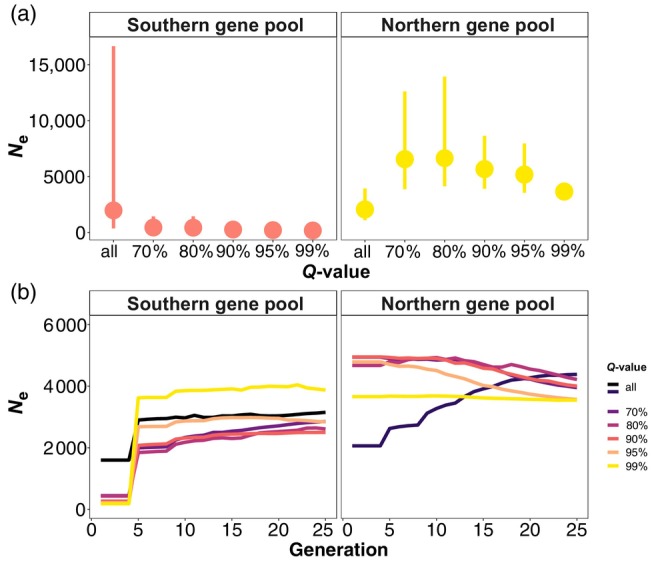
Influence of population structure on the *N*_e_ estimates for the Northern and Southern gene pools of *Prunus armeniaca*, as obtained with GONE. *Q*‐values refer to the results of the fastStructure analysis performed in Groppi et al. (2021) (lower bounds of individual *Q*‐value to the main genetic cluster). *N*_e_ was estimated over 50 datasets of resampled individuals (77 in each *Q*‐value subset in the Northern gene pool and 21 in each *Q*‐value subset in the Southern gene pool, reflecting differences in sample sizes). *N*_e_ estimates for the combined gene pools are also shown (“all”), obtained by resampling individuals (77 individuals when compared with the Northern gene pool estimates and 21 individuals when compared with the Southern gene pool estimates). In (a), points represent the geometric mean and ranges represent 95% confidence intervals across 50 replicates; in (b), only geometric mean values of the *N*_e_ estimates across 50 replicates and in the last 25 generations are shown.

The impact of admixture on *N*_e_ estimation was explored using the dataset of *P. armeniaca*. Estimates of *N*_e_ in the most recent generation generally decreased when the *Q*‐value of the individuals included in the analysis increased (Figure 5a). The larger *N*_e_ estimates in the most recent generations (1–4) when including more admixed individuals are consistent with the upward bias predicted by Waples and England (2011) for a sampled subpopulation that does not include all potential parents (“drift LD”); with higher admixture proportions (Figure 5a), the *N*_e_ estimated for each gene pool (subpopulation) using the LD method tends to approach the *N*_e_ of the metapopulation instead (Waples & England, 2011). However, the *N*_e_ estimate we obtained when combining the two gene pools (“all” in Figure 5a) was lower than the *N*_e_ estimate obtained when considering highly admixed individuals in the Northern gene pool (70% in the right panel of Figure 5a). A downward bias in the *N*_e_ estimation is expected because of the Wahlund effect associated with sampling and analysing different gene pools together (“mixture LD”; Neel et al., 2013; Nunney, 2016; Waples, 2024; Waples & England, 2011). Using simulations, Novo, Ordás, et al. (2023) demonstrated that both the time of gene pool divergence and the timing of the mixing event may affect the bias in the *N*_e_ estimation. The longer the time elapsed since the gene pools diverged, the more pronounced the downward bias on *N*_e_ becomes. Similarly, the more recent the mixing event (in our case, as a consequence of sampling strategy), the more exacerbated the downward bias on *N*_e_. If the occurrence of a mixing event is unknown, the decrease in *N*_e_ might mistakenly be interpreted as a reduction in population size, such as that caused by a bottleneck.

The Southern gene pool showed a contrasting trend; *N*_e_ estimates for the less admixed groups remained lower than that obtained when combining the two gene pools, possibly because the few samples from this gene pool contributed less (with any potential mixture LD) than the more abundant samples from the Northern gene pool (with their LD signal) (Figure 5a). However, the large confidence intervals might also suggest a combined effect of drift LD and bias in the estimates induced by using a small sample (21 individuals) to estimate a large *N*_e_ (of the metapopulation). How the relationship between sampling and genetic structure practically affects *N*_e_ still deserves evaluation, as the effect on LDNe will depend on the relative strength of the “mixture LD” and the “drift LD” in the specific set of samples included in the analyses (Waples, 2024).

Over the last 25 generations (Figure 5b), we obtained higher *N*_e_ estimates when individuals from the Southern gene pool with a *Q*‐value ≥ 99% were included. For the Northern gene pool, on the contrary, we obtained a lower *N*_e_ estimate when individuals with a *Q*‐value ≥ 99% were included. The different demographic histories of the Northern and Southern gene pools certainly underlie the pattern observed, as the Southern gene pool seems to have undergone a recent bottleneck, whereas the Northern gene pool has a more stable demographic trend. The recent population decline for the Southern gene pool may be explained by the Soviet era and the current land‐use change in the Fergana valley (mainly Uzbekistan) where native forests of wild apricot were partially replaced with crop species. Nevertheless, two more factors should be considered; first, the sample size of the Southern gene pool is smaller than that of the Northern gene pool (only 21 individuals vs. 77 individuals drawn from each *Q*‐value subset). Second, Santiago et al. (2020) warn about a typical artefactual bottleneck observed in GONE and caused by population structure (in figure 2F of Santiago et al., 2020, considering a migration rate = 0.2%; Novo, Ordás, et al., 2023). As we observed a consistent trend regardless of the individual *Q*‐value, and the drop in *N*_e_ is particularly evident with a *Q*‐value = 99%, we interpret this *N*_e_ drop as a true bottleneck, with the caveat of reduced accuracy linked to a small sample size for the Southern gene pool.

#### 3.1.5 Effect of using genomic scaffolds rather than chromosomes

**FIGURE 6:**
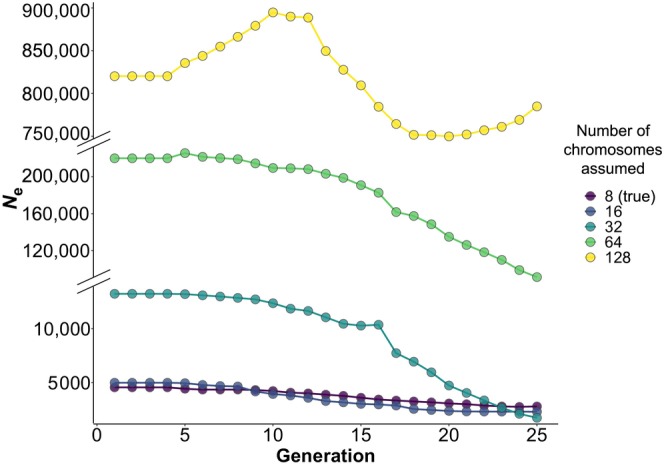
Estimates of *N*_e_ calculated on datasets in which the same set of SNPs is assigned to a progressively larger number of assumed chromosomes, where 8 is the true number of chromosomes for *Prunus armeniaca* (per haploid count); 45 individuals from the Northern gene pool were used for this analysis.

To evaluate the effect of using genomic scaffolds as a proxy for linkage groups when chromosome information is not available, we sorted SNPs from the *P. armeniaca* dataset into a progressively larger number of scaffolds or chromosomes assumed. This produced inconsistent *N*_e_ estimates across the datasets with increasing number of chromosomes assumed, with *N*_e_ values progressively rising from around 3 × 10^3^ for 8 chromosomes (true value) to >8 × 10^5^ when the number of chromosomes assumed was equal to 128 (Figure 6). The algorithm implemented in GONE is based on the assumption that LD among pairs of SNPs at different genetic distances provides differential information about *N*_e_ at different times in the past (Santiago et al., 2020). Loosely linked loci give information about *N*_e_ in recent generations, as their recombination rate is higher and rate of LD‐decay slower than that of closely linked loci (Sved & Feldman, 1973). Therefore, the behaviour of the *N*_e_ estimates observed in Figure 6 can be explained if considering that when a chromosome is broken into smaller scaffolds, only closely linked loci will be available for the *N*_e_ estimation; pairs of SNPs at higher genetic distances (i.e., loosely linked loci) will be missing, inducing biases on recent *N*_e_ estimates. An inflated *N*_e_ in recent generations will therefore depend on having fewer random associations among loci useful to estimate LD (i.e., fewer loosely linked loci), which will unfold as having less genetic drift (i.e., a larger population). Consequently, *N*_e_ estimates obtained with GONE for *M. annua* and *F. sylvatica* may be biased upward since scaffolds were used as a proxy for chromosomes (Table 1).

### 3.2 *N*_e_ estimates obtained with GONE, NeEstimator and *currentNe*

As expected, *N*_e_ estimates obtained using NeEstimator and *currentNe* were more in agreement with one another compared with those obtained with GONE for the last generations (Table 2). GONE estimates for all species were larger than those obtained using the other programs, especially in the Northern gene pool of *P. armeniaca* (GONE‐*N*_e_ ≃ 3500 for the last generation while NeEstimator‐*N*_e_ ≃ 716.2, excluding singletons and after bias correction, and *currentNe‐N*_e_ ≃ 170). The point *N*_e_ estimate obtained with *currentNe* and its confidence intervals remained consistent even when we increased the number of SNPs, suggesting that there was no uncertainty associated with the SNPs included in the analysis. Estimates from simulated populations in Santiago et al. (2024) showed consistency between the output of *currentNe* and NeEstimator, except when a small sample (10 individuals) was drawn from a very large population (*N*_e_ = 10,000) using 22,000 SNPs, in which case *currentNe* performed better. Our sample size for the Northern gene pool was much larger (77 individuals), and we do not expect the true *N*_e_ to be larger than 10,000. Therefore, when using the same dataset for *currentNe* and NeEstimator, we interpret the slight discrepancy between the two estimates to be associated with the different algorithms included in the programs, which are affected in different ways by the occurrence of rare alleles and the deviations from random mating, among other things (Santiago et al., 2024). When considering the Southern gene pool, for which the true *N*_e_ is expected to be smaller than for the Northern gene pool (Groppi et al., 2021), the estimates obtained with GONE (184) was higher than those obtained with NeEstimator (80.9 excluding singletons and after bias correction) and *currentNe* (≃30).

**TABLE 2:** Estimates of effective population sizes for each dataset analyzed in GONE, NeEstimator, and *currentNe*.

Species gene pool (#samples)	*N*_e_ in GONE		*N*_e_ in NeEstimator			*N*_e_ in *currentNe*	
	#polymorphic loci^a^	*N*_e_ ^b^	#polymorphic loci^c^	*N*_e_ (95% CI) ‐ excluding singletons^d^	*N*_e_ (95% CI) ‐ no MAF filtering	#polymorphic loci	*N*_e_ (90% CI)^e^
*S. globulifera* Species 1 (228)	17,515	N/A	17,515	754 (623–949)	1036 (841–1340)	N/A	N/A
Species 2 (107)	14,906	N/A	14,906	380 (300–510)	547 (409–813)	N/A	N/A
Species 3 (30)	9207	N/A	9207	86 (37‐Inf)	223 (65‐Inf)	N/A	N/A
*M. annua* Atlantic (12)	17,854	40	17,854	15 (7–58) 27.3, after correction^f^	22 (10–121) 40, after correction^f^	17,854	17.6 (13.3–23.3)
Core (16)	27,874	123	27,874	18.6 (10.2–46.2) 33.8, after correction^f^	34.7 (18.3–131.3) 63.1, after correction^f^	27,874	20.4 (16.2–25.7)
Mediterranean (12)	18,032	103	18,032	16 (10–32) 29.1, after correction^f^	26 (17–51) 47.3, after correction^f^	18,032	20.5 (15.2–27.6)
*F. sylvatica* (35)	322,185 (12 scaffolds)1,115,200 (27 scaffolds)	25 (12 scaffolds) 360 (27 scaffolds)	41,103 (12 scaffolds)	1.5 (1.1–1.4) 2.3, after correction^f^	1.1 (0.8–0.9) 1.7, after correction^f^	1,238,257 (12 scaffolds)	4.0 (5.0–5.0)
*P. armeniaca* Southern (21)	82,891	184	11,559	44.5 (34.5–61.3) 80.9, after correction^f^	71.2 (55.6–97.4) 129.5, after correction^f^	333,829 (subset with 1.5 million SNPs)	30 (22.9–39.5)
						11,120 (subset with 50,000 SNPs, as in NeEstimator)	27.6 (20.0–38.0)
Northern (77)	116,285	3526	16,100	393.9 (252.8–838.6) 716.2 after correction^f^	510.2 (311.3–1309.5) 927.3 after correction^f^	444,946 (subset with 1.5 million SNPs)	171.4 (144.1–203.9)
						17,794 (subset with 50,000 SNPs, as in NeEstimator)	170.3 (138.0–210.1)

^a^ Number of polymorphic loci analyzed in each program. GONE only uses a subset of SNPs per chromosome (or scaffold), up to a maximum of 50,000 SNPs per chromosome (or scaffold), these are indicated in the OUTPUT_dataname file.

^b^ *N*_e_ in GONE for the last generation (geometric mean); no MAF filtering was applied, as recommended.

^c^ Note that in NeEstimator and in *currentNe*, SNPs = loci. Polymorphic loci in NeEstimator = total number of loci minus number of non‐polymorphic loci.

^d^ As low‐frequency alleles upwardly bias *N*_e_, we followed the recommendations in Waples (2024) and excluded singleton alleles. CIs in NeEstimator represent jackknife confidence intervals.

^e^ *N*_e_ estimation by integration over the whole genome as output by *currentNe*, except in *P. armeniaca*, where SNPs mapping was available and the “*N*_e_ estimation based on LD between chromosomes” was used.

^f^ When the information about the number of chromosomes was available, estimates obtained with NeEstimator were corrected using (*N*_e_ estimate)/*y*, where *y* represents the formula in Waples et al., 2016: *y* = 0.098 + 0.219 × ln(*Chr*), with *Chr* as the (haploid) number of chromosomes; *M. annua*: 8 chromosomes, *F. sylvatica*: 12 chromosomes, *P. armeniaca*: 8 chromosomes.

Another consideration is the downward bias on *N*_e_ estimates caused by localized sampling in continuous populations featuring isolation by distance (Neel et al., 2013; Nunney, 2016; Santos‐del‐Blanco et al., 2022; Waples, 2024). If the range of sampling is similar in extent to the unknown effective range of dispersal, as it is likely the case in *S. globulifera*, estimates may not reflect the population‐wide true *N*_e_, but rather a quantity close to the neighbourhood size (*N*_s_), i.e., the inverse of the probability of identity by descent of two uniting gametes (Santos‐del‐Blanco et al., 2022). In *P. armeniaca*, where the sampling window likely exceeded the breeding window by much, we may still expect a downward bias because of the mixture LD caused by the inclusion of genetically divergent individuals (Neel et al., 2013; Waples, 2024; Waples & England, 2011). However, this bias would not explain the discrepancy between the estimates obtained with GONE and those obtained with the other programs for the Northern gene pool of *P. armeniaca*. In *S. globulifera*, for which we also expect a large *N*_e_ (>1000), it was only possible to use NeEstimator, due to the short length of contigs (not appropriate when using GONE), and the lack of information about the number of chromosomes (as required to obtain reliable estimates with *currentNe*). *N*_e_ ranged from 86 (CI: 37‐Infinite) in Species 3, to 380 (CI: 300–510) in Species 2 and to 754 (CI: 623–949) in Species 1, although point estimates could not be corrected for physical linkage due to lack of information about chromosome number and are therefore biased downward (Table 2). Estimates for Species 3, in particular, displayed infinite confidence intervals, suggesting that the sample size might be not large enough to capture the genetic drift signal from the original population. However, the relative magnitude of the estimates obtained are in agreement with the availability of suitable habitats for the three species (Schmitt et al., 2021) and, all else being equal, we would generally expect these populations to have a long‐term constant population size, considering that the Guianese rainforest has experienced a continuous forest cover since the last glacial maximum (Barthe et al., 2017).

The uncertainty in *N*_e_ estimation using the LD method is particularly exacerbated in the dataset from *F. sylvatica* where, in addition to the potential downward bias induced by localized sampling in a continuous population, missing data also affect the estimation performed with the three programs (GONE‐*N*_e_ = 25 for the last generation, NeEstimator‐*N*_e_ = 2.3, excluding singletons and after bias correction for physical linkage, and *currentNe*‐*N*_e_ = 4 after bias correction for physical linkage), by reducing the usable sample size among pairs of loci (Do et al., 2014; Peel et al., 2013; Waples, 2024). In general, missing data affect the precision of *N*_e_ estimates from the LD method whereas accuracy should be less affected (Nunziata & Weisrock, 2018; Waples, 2024), unless missing data occur non‐randomly and depend on the genotype, as it might be the case in the *F. sylvatica* dataset.

For the only annual plant in our dataset, *M. annua*, we would expect *N*_e_ estimated with the LD method to mainly reflect the effective number of breeders, *N*_b_ (Luikart et al., 2021; Waples, 2024) for the year of sampling, as individual cohorts were sampled (progeny of adults that reproduced in that specific year). Estimates from GONE were higher than those obtained with NeEstimator and *currentNe* (Table 2), also because of the bias induced by the lack of SNPs mapping (i.e., using scaffolds as a proxy for chromosomes in GONE). All point estimates fell within the estimated confidence intervals and usually denoted a small *N*_e_, which is consistent with primarily reflecting the *N*_b_ for the population. In particular, point estimates in NeEstimator, excluding singletons and after bias correction for physical linkage, ranged from 29.1 for the Mediterranean gene pool to 33.8 for the Core gene pool and 27.3 for the Atlantic gene pool. Point estimates in *currentNe* ranged from 20.5 for the Mediterranean gene pool to 20.4 for the Core gene pool and 17.6 for the Atlantic gene pool. Even if the gene pool subdivision was consistent with the level of genetic admixture found in the individuals, it is still possible that estimates are biased downward because of mixture LD associated with mixing samples from different geographical locations (sampling window larger than breeding window). Furthermore, *M. annua* is able to survive through multi‐annual seed banks (Crocker, 1938) despite being an annual plant, and therefore the arithmetic mean across multigenerational *N*_b_ estimates would be needed to reliably estimate *N*_e_ rather than *N*_b_ (Nunney, 2002; Waples, 2006b).

### 3.3 Practical recommendations when estimating contemporary *N*_e_ in GONE

In this study, we have considered some of the technical limitations when estimating *N*_e_ from plant genomic datasets, including: (i) the occurrence of missing data, (ii) the limited number of SNPs/individuals sampled, and (iii) the lack of genetic/linkage maps and of information about how SNPs map to chromosomes when estimating *N*_e_ using the software GONE. In addition, we have explored some biological limitations that may affect *N*_e_ estimation using the LD method, such as the occurrence of population structure, although we recognize that our exploration is not exhaustive, as other biological factors (i.e., associated with reproductive system and life‐history traits) might affect *N*_e_ and its estimation. Our empirical results corroborate some previous findings (reviewed in Waples, 2024) about the importance of having large samples sizes, especially when populations are large. For example, we found that >30 individuals were necessary to reach consistent *N*_e_ estimates (≃several thousands) for *P. armeniaca*. Furthermore, our empirical results highlight the following requirements that genomic datasets should satisfy:

- non‐random missing data should not exceed 20% per individual. Missing data also affect how SNPs are represented across loci and individuals sampled and can generate non‐random patterns whose effect on *N*_e_ estimation is difficult to predict (as observed in the *F. sylvatica* and *P. armeniaca* datasets);
- having a large number of SNPs (>tens of thousands) is potentially important to allow users to generate non‐overlapping subsets of loci that reduce the influence of pseudoreplication on confidence intervals (Waples et al., 2022). However, increasing the number of SNPs beyond a few thousands per chromosome does not produce significant changes in the *N*_e_ estimates, as we observed in wild apricots; Waples (2024) also observed that the benefit of adding over a few thousand SNPs on precision is little, but increases if the true *N*_e_ is very large.
- most importantly, having SNPs fully mapped to chromosomes is essential to obtain reliable estimates when using the software GONE (as observed in the *P. armeniaca* dataset); other programs should be preferred to estimate contemporary *N*_e_ when SNPs mapping is not available (i.e., *currentNe*).

In addition, the bias on *N*_e_ estimates due to the occurrence of gene flow and admixture can significantly affect the performance of single‐sample estimators (as observed in the *P. armeniaca* gene pools), as previously described (e.g., Neel et al., 2013). Other biases associated with (i) further sources of population structure (i.e., overlapping generations, demographic fluctuations including bottlenecks, reproductive strategies causing variance in reproductive success, etc.) and (ii) further technical issues associated with sampling strategies and genomic datasets can add up and generate results that are misleading for conservation. Therefore, a careful consideration of the issues above is essential when designing and interpreting studies focused on the estimation of *N*_e_ and other related indicators for conservation.

## CONFLICT OF INTEREST STATEMENT

The authors have no conflict of interest to declare.

## BENEFITS GENERATED STATEMENT

Benefits from this research accrue from the sharing of our data and results on public databases as described above.

## Supporting information


File S1

File S2

Table S1

## Data Availability

The SNP matrices used in this study can be accessed at the following links: https://doi.org/10.5281/zenodo.4727831 (*Symphonia globulifera*; Schmitt et al., 2021), https://datadryad.org/stash/dataset/doi:10.5061/dryad.74631 (*Mercurialis annua*; González‐Martínez et al., 2018), https://doi.org/10.57745/FJRYI1 (*Fagus sylvatica*; Lesur Kupin & Scotti, 2023), https://doi.org/10.5281/zenodo.8124822 (*Prunus armeniaca*; Gargiulo et al., 2023). The analyses carried out in this study and the related scripts are available at: https://github.com/Ralpina/Ne‐plant‐genomic‐datasets (Gargiulo, 2023).
