# Supplementary material for: Estimation of contemporary effective population size in plant populations: Limitations of genomic datasets
Source: Evol Appl. 2024 May 3;17(5):e13691. doi: 10.1111/eva.13691 (PMC11069024; doi:10.1111/eva.13691)
Supplement: Supplementary file 3 — Table S1 [file EVA-17-e13691-s003.docx]

**Supplementary Table 1.** Groups of *Prunus armeniaca* based on Q-values as obtained in Groppi et al. 2021. See also: <https://doi.org/10.5281/zenodo.8124822>

| **Gene pool** | **fastStructure Q-value assignment (Groppi et al. 2021)** | **Initial sample sizes from which resampled individuals were obtained** |
| --- | --- | --- |
| Northern | Individuals with Q-value > 99% | 77 |
| Northern | Individuals with Q-value > 95% | 126 |
| Northern | Individuals with Q-value > 90% | 164 |
| Northern | Individuals with Q-value > 80% | 189 |
| Northern | Individuals with Q-value > 70% | 199 |
| Southern | Individuals with Q-value > 99% | 21 |
| Southern | Individuals with Q-value > 95% | 33 |
| Southern | Individuals with Q-value > 90% | 38 |
| Southern | Individuals with Q-value > 80% | 50 |
| Southern | Individuals with Q-value > 70% | 56 |
| All | Individuals from both gene pools | 255 |
